# Tracking a misclassified pathogen: genomic and epidemiological features of Vibrio paracholerae

**DOI:** 10.1099/mgen.0.001605

**Published:** 2026-01-12

**Authors:** Sergio Mascarenhas Morgado, Erica Lourenço da Fonseca, Ana Carolina Paulo Vicente

**Affiliations:** 1 Instituto Oswaldo Cruz, Laboratório de Genética Molecular de Microrganismos, Av. Brasil, 4365 - Manguinhos, Rio de Janeiro, Brazil

**Keywords:** antibiotic resistance gene (ARG) reservoir, *bla*CARB, non-O1/O139 *Vibrio cholerae*, *qnr*VC, sedentary integron, superintegron

## Abstract

The genus *Vibrio* encompasses globally relevant pathogens, of which *Vibrio cholerae* is the best known due to its role in cholera. Closely related species within the Cholerae clade *– Vibrio paracholerae*, *Vibrio metoecus* and *Vibrio tarriae* – were long misclassified as non-O1/O139 *Vibrio cholerae*. The objective of this study was to analyse all 13,000+ available *V*. *cholerae* genomes in GenBank to determine the presence of species from the Cholerae clade. Genome-wide analyses using Mash, whole-genome-based Average Nucleotide Identity and digital DNA–DNA hybridization reclassified 190 unique genomes as *V. paracholerae*, while *V. metoecus* and *V. tarriae* were not detected. Phylogenomic analyses revealed that *V. paracholerae* forms distinct lineages, spanning clinical, environmental and animal sources over a period of more than a century. Virulence profiling revealed the absence of cholera toxin and toxin-coregulated pilus; however, most genomes exhibited other virulence factors, including haemolysins, RTX toxins, cholix toxin and a conserved type VI secretion system. Resistome analysis revealed multiple antibiotic resistance genes, several of which were embedded within superintegron regions, reinforcing the role of *V. paracholerae* as a reservoir of resistance determinants. Importantly, we identified five putative gene markers with high sensitivity and specificity for discriminating the two species, providing a tool for diagnostic applications and epidemiological surveillance. These findings reveal an unsuspected epidemiological scenario for *V. paracholerae*, which should be considered in clinical monitoring and public health strategies involving the Cholerae clade.

**Impact Statement**

This study provides a comprehensive genomic reassessment of *Vibrio paracholerae*, the closest sister species of *Vibrio cholerae*, revealing that nearly 2% of genomes previously classified as *V. cholerae* are in fact *V. paracholerae*. This misidentification, detected in both clinical and environmental isolates, may introduce taxonomic and epidemiological bias, with direct implications for disease surveillance, outbreak investigations and treatment strategies. By clarifying species boundaries through genomic and phylogenomic approaches, and by identifying highly discriminatory molecular markers, this work delivers practical tools for accurate *V. paracholerae* identification by means of genomic and/or routine diagnosis. Furthermore, the discovery that *V. paracholerae* persists across diverse ecological niches, while acting as a reservoir for antibiotic resistance genes, highlights its underestimated role in microbial ecology and public health. These findings advance our understanding of *Vibrio* evolutionary dynamics and provide resources for improving diagnostic accuracy and genomic epidemiology of cholera-related pathogens.

## Data Summary

The authors confirm that all supporting data, code and protocols have been provided within the article or through supplementary data files.

## Introduction

The genus *Vibrio* encompasses globally relevant species that are common etiological agents of diseases in humans and aquatic organisms, with *Vibrio cholerae* being the most well-known as the causative agent of cholera [1]. The pathogenicity of certain *V. cholerae* lineages is largely attributable to specific virulence determinants, including the cholera toxin (CTX) and the toxin-coregulated pilus (TCP) [2], which have also been sporadically reported in other *Vibrio* species [3].

In recent years, some species closely related to *V. cholerae* have been identified, most of which were initially misclassified as non-O1/O139 *V. cholerae*. These members of the Cholerae clade include *Vibrio paracholerae* [4], *Vibrio metoecus* [5] and *Vibrio tarriae* [6]. Although the pathogenic potential of these *V. cholerae*-related species varies, they have also been associated with human infections [7]. These species appear to coexist with both environmental and clinical *V. cholerae* lineages, some of which are linked to epidemics and pandemics and harbour major cholera virulence determinants such as CTX and VPI-1 [8]. This ecological overlap provides an ideal context for the exchange of genetic material, including genes encoding virulence factors [3,4, 9], thereby facilitating the emergence of toxigenic *Vibrio*.

Specifically, *V. paracholerae* has been described as the closest known sister species to *V. cholerae*, based on genetic and genomic analyses [4], and has historically been associated with human infections, with reports dating back to 1916 [10]. This species has been linked to diarrhoea, bacteraemia and sepsis [11]. Consequently, its misidentification may introduce bias into epidemiological analyses and hinder appropriate treatment and disease control strategies.

In this study, we conducted a comprehensive genomic and phylogenetic reassessment to clarify the boundaries between *V. cholerae* and other closely related *Vibrio* species, particularly *V. paracholerae*. Using phylogenomics, digital DNA–DNA hybridization (dDDH) and whole-genome Average Nucleotide Identity (gANI), we established reliable cutoffs for species delineation and proposed potential molecular markers.

## Methods

### Genome collection and initial screening

A total of 13,206 *V*. *cholerae*, 69 *V*. *metoecus*, 62 *V*. *paracholerae* and 30 *V*. *tarriae* genomes were retrieved from GenBank (accessed April 2025). Since 30 out of 62 *V*. *paracholerae* genomes corresponded to redundant assemblies, they were removed, and only 32 out of 62 genomes were retained for further analysis. To identify potentially misclassified genomes within the 13,206 *V*. *cholerae* assemblies, we next selected representative reference genomes from closely related species *– V. metoecus* ZF102, *V. paracholerae* NCTC 30 and *V. tarriae* 2521-89. Pairwise genomic distances were estimated using Mash v2.3, which applies a MinHash sketching approach to approximate average nucleotide identity [12]. Mash distances were calculated between each reference genome and the 13,206 *V*. *cholerae* assemblies. Genomes presenting higher shared-hash values (i.e. lower Mash distances) with *V. metoecus*, *V. paracholerae* or *V. tarriae* references were flagged as candidates for potential misclassification. These genomes were retained for subsequent high-resolution analyses, including gANI and dDDH, to confirm species boundaries and refine taxonomic assignments. Genome quality (completeness and contamination) was evaluated using CheckM2 v1.1.0 (https://github.com/chklovski/CheckM2), where genomes presenting completeness ≥90% and contamination ≤10% were retained.

### Genomic similarity and phylogenetic analyses

Genomes identified in the Mash-based screening as potential misclassifications were subjected to high-resolution similarity and phylogenetic analyses. Alignment fraction (AF) and gANI were computed using MiSI (Microbial Species Identifier) [13], which applies genome-wide pairwise blast-based comparisons. Species delineation followed the recommended thresholds of AF ≥0.6 and gANI ≥96.5%. In parallel, dDDH values were estimated using the Genome-to-Genome Distance Calculator (GGDC 3.0) with the recommended *formula 2* (identities/HSP length), which provides a robust correlation with traditional wet-lab DNA–DNA hybridization [14].

For phylogenomic reconstruction, we first identified core genes across the dataset using Roary v3.13.0 [15], with a minimum sequence identity of 95% for orthologous clustering. The resulting core gene alignment was processed with snp-sites v2.5.1 (https://github.com/sanger-pathogens/snp-sites) to extract informative SNPs. Maximum-likelihood phylogenetic trees were then inferred with IQ-TREE v2.4.0 [16], employing the best-fitting nucleotide substitution model selected by ModelFinder and 1,000 ultrafast bootstrap replicates to assess nodal support. Final trees were visualized and annotated using iTOL v6 [17]. Roary was also used for pangenome analysis, in which genes highly prevalent among *V. paracholerae* genomes were queried against the *V. cholerae* dataset using BLASTn to identify eventual unique *V. paracholerae* genes to be used as markers.

### Detection of resistance and virulence genes

Antibiotic resistance genes (ARGs) and virulence-associated genes were screened using the abricate tool (https://github.com/tseemann/abricate). Searches were performed against the Comprehensive Antibiotic Resistance Database for ARGs and the Virulence Factor Database (core dataset) for virulence factors. A minimum threshold of ≥80% sequence identity and ≥80% coverage was applied for gene detection.

To investigate the genomic context of resistance determinants, we further examined the flanking regions of each identified ARG. Specifically, 500 bp of sequences was extracted from both upstream and downstream regions and searched against Vibrio chromosomal repeats (VCRs) using BLASTn. The search considered sequences with >50 % identity and >70 % alignment coverage.

## Results and discussion

Accurate bacterial species identification, especially for those involved in epidemics and pandemics, is crucial for understanding epidemiological dynamics, guiding effective control measures and improving the clinical management of infections.

### Reclassification of *V. cholerae* genomes as *V. paracholerae*

To assess potential misclassified *V. cholerae* genomes, we retrieved all available assemblies from GenBank (*n*=13,206) and compared them with representative genomes from other members of the Cholerae clade. Pairwise genomic distances were first estimated using Mash, with *V. paracholerae*, *V. metoecus* and *V. tarriae* reference genomes as queries against the *V. cholerae* dataset. Genomes exhibiting the highest shared hashes (≥100 out of 1,000) were retained for further analysis using AF and gANI. By these methods, no *V. cholerae* genome exceeded the threshold values required for species-level assignment to either *V. metoecus* or *V. tarriae*. However, several candidate genomes displayed gANI values consistent with *V. paracholerae* and were therefore selected for more detailed comparative analyses.

Using *V. paracholerae* NCTC 30 as reference, we identified a preliminary subset of 523 out of 13,206 *V*. *cholerae* genomes that shared ≥330 out of 1,000 hashes in the Mash analysis and retained them for downstream comparison. As a baseline, we first calculated the AF and gANI values for the 32 genomes previously recognized as *V. paracholerae*, using *V. cholerae* N16961 as reference. These genomes showed AF values ranging from 0.83 to 0.93 and gANI values ranging from 95.76% to 96.34% (Table S1, available in the online Supplementary Material). Although all AF values exceeded the ≥0.6 cutoff and therefore could not discriminate between the two species, the gANI values fell below the 96.5% threshold for species delineation. For this reason, gANI was used as the primary metric to compare the two species. Subsequently, we computed gANI values for the putative set of 523 candidate *V. paracholerae* genomes, using *V. paracholerae* NCTC 30 as reference. Among them, 238 genomes, originally annotated as *V. cholerae*, exceeded the 96.5% gANI threshold, suggesting their reassignment to *V. paracholerae* (Table S1). Analyses of these 238 genomes revealed that 48 represented redundant assemblies, which were subsequently removed from downstream analyses, leaving a total of 190 unique genomes (Table S1). These genomes were then subjected to dDDH analysis using *V. cholerae* N16961 as reference. The resulting dDDH values ranged from 64.5% to 68.7% (Table S1), all below the 70% threshold for species delineation, providing further support for their reassignment to *V. paracholerae*. Applying dDDH to the putative 190 *V*. *paracholerae* genomes, using *V. paracholerae* NCTC 30 as the reference, yielded values ranging from 74% to 100% (Table S1), corroborating our previous analysis.

**Fig. 1.**
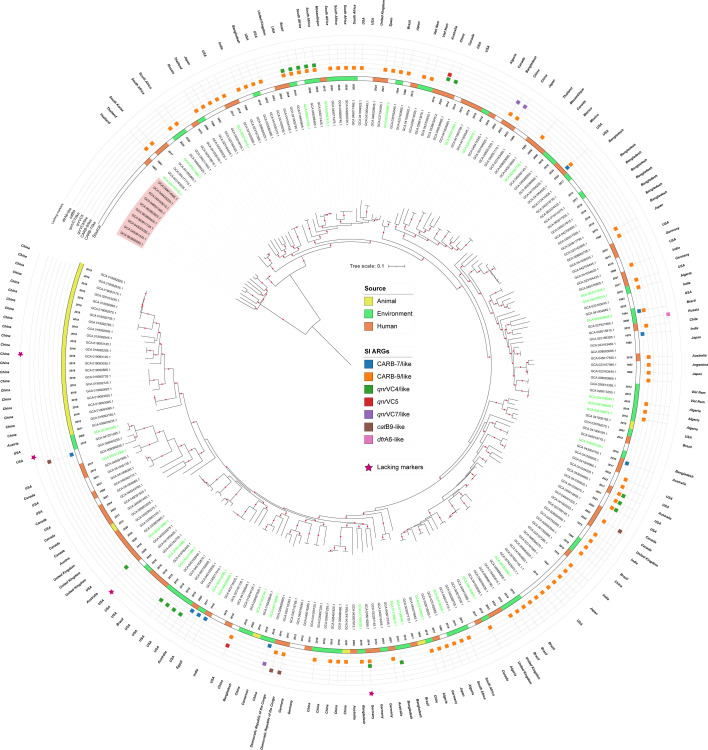
Maximum-likelihood core genome SNP phylogeny of *Vibrio* genomes. Two *Vibrio* species are shown: *V. cholerae* (red background) and *V. paracholerae* (uncoloured background). Coloured strips adjacent to the labels indicate the isolation source of each genome. Genomes labelled in green represent reference *V. paracholerae* genomes. Coloured squares denote the presence of ARGs within the superintegron (SI) of the corresponding genome. Dark magenta stars indicate *V. paracholerae* genomes lacking the five markers. Genomes without labels correspond to entries with missing metadata in their BioSample records. Red circles on branches represent >70% bootstrap.

Finally, the complete set of 222 *V*. *paracholerae* genomes (32 already identified as *V. paracholerae* in GenBank plus 190 reclassified/this study) was subjected to phylogenomic analysis based on the core genome of the dataset. The resulting phylogenetic tree clearly separated *V. paracholerae* from *V. cholerae* (Fig. 1), in agreement with the gANI and dDDH results. These analyses demonstrate that misidentification of *V. paracholerae* as *V. cholerae* occurred in ~1.8% of all *V. cholerae* genomes, highlighting a previously underappreciated source of taxonomic and epidemiological bias.

### *V. paracholerae* genomic epidemiology

The phylogenomic analysis of the 222 *V*. *paracholerae* genomes revealed that they belonged to distinct lineages (Fig. 1). Metadata associated with these genomes (when available) indicated their sources as clinical settings (*n*=69), environmental samples (*n*=59) and animals (*n*=28). Interestingly, some lineages encompassed isolates collected from different sources and across wide temporal and geographic ranges. For example, one lineage includes isolates obtained more than a century apart, from Egypt in 1916 (clinical; GCA_900538065.1) and the USA in 2017 (environmental; GCA_003312095.1). Other noteworthy clusters highlight this diversity: GCA_041005155.1 (USA/2019/animal) and GCA_035782075.1 (Brazil/2001/environment); GCA_045016505.1 (Germany/2021/clinical) and GCA_024105925.1 (Algeria/2018/environment); GCA_001402745.1 (Bangladesh/2002/environment) and GCA_016456645.1 (China/2015/animal); GCA_006803035.1 (Austria/2011/zooplankton) and GCA_019093165 (China/2018/migratory birds). Notably, two *V. paracholerae* genomes (GCA_030710345.1 and GCA_030718785.1) were recovered in the Democratic Republic of the Congo in the context of the 2009–2012 cholera outbreak, coinciding in time and location with the epidemic *V. cholerae* lineages AFR10d and AFR10e [8]. Taken together, these findings demonstrate that *V. paracholerae* lineages are able to persist across space and time, occurring in the environment (sometimes alongside *V. cholerae*), in clinical contexts and in animal hosts, highlighting their ecological versatility and clinical and epidemiological relevance.

### Identification of species-specific markers for *V. paracholerae*

For epidemiological tracking purposes, it is important to establish reliable markers capable of discriminating between *V. cholerae* and *V. paracholerae*. A previous study by Islam *et al.* (2021) identified two genes: *LysR* family transcriptional regulator (WP_001924807.1) and *HAD-IB* family hydrolase (WP_071179638.1); reported to be present in 22 *V*. *paracholerae* strains and absent from 22 *V*. *cholerae* strains. Screening these genes throughout our dataset (222 *V*. *paracholerae* and 12,946 *V*. *cholerae* genomes), 4 *V. paracholerae* genomes lacked both genes (GCA_019093125.1, GCA_041005405.1, GCA_045016285.1 and GCA_047251285.1), whereas 27 *V*. *cholerae* genomes harboured them. Accordingly, the two markers exhibited 98% sensitivity (218 out of 222 *V*. *paracholerae*) and >99.99% specificity (27 out of 12,946 *V*. *cholerae*). These findings prompted us to investigate whether additional markers could be identified using this larger dataset.

Based on the *Roary* pangenome results, orthologous groups were defined, and those highly prevalent in *V. paracholerae* were screened against *V. cholerae* using both BLASTn and abricate. This approach revealed five putative genetic markers, including the two previously described, that were present in 218 of the 222 *V*. *paracholerae* genomes (Table 1).

**Table 1.** Putative species-specific markers for *V. paracholerae*

Gene	Size (bp)	Number of *V. paracholerae*	Number of *V. cholerae*	Reference locus tag*	Product	Prevalence of *V. cholerae*
*ser*B	663	218	27	SAMEA104470976_01923	HAD-like hydrolase superfamily SerB	0.002%
*gcv*A	894	218	27	SAMEA104470976_01924	LysR-type transcriptional regulator	0.002%
–†	1020	218	25	SAMEA104470976_01925	Aminoethylphosphonate-binding protein ABC transport system	0.0019%
*pot*A	1062	218	25	SAMEA104470976_01926	Aminoethylphosphonate ABC transport system, ATP-binding component PhnT2	0.0019%
*pot*H	1716	218	25	SAMEA104470976_01927	Aminoethylphosphonate ABC transport system, permease	0.0019%

*V. paracholerae strain NCTC 30: LS997867.1.

†Gene acronym was not identified.

The four *V. paracholerae* genomes missing the five markers were GCA_019093125.1, GCA_041005405.1, GCA_045016285.1 and GCA_047251285.1. These genomes exhibited 100% genome completeness (Table S1) and did not cluster together in the phylogeny (Fig. 1, dark magenta stars), supporting the conclusion that this genomic region is genuinely absent rather than missing due to technical artefacts. Interestingly, the five markers are arranged as a contiguous 5,930 bp locus (SAMEA104470976_01923 to SAMEA104470976_01927), consistent with a block deletion event in these four genomes. Despite their broad distribution, these genes showed modest sequence variation, with nucleotide identities ranging from 97.74% to 100 %, indicating strong conservation within *V. paracholerae*.

Since these markers are highly prevalent in *V. paracholerae* and nearly absent from *V. cholerae*, their distribution is consistent with horizontal gene transfer, a process previously proposed between these species [4]. Indeed, in the few *V. cholerae* genomes where these genes were detected, their presence suggests a singular acquisition event. However, no signatures of mobile genetic elements were identified in the regions flanking this locus. Overall, these markers displayed high sensitivity (98%) and specificity (>99 %), reinforcing their potential as species-discriminating genetic targets for routine laboratory applications.

### Virulence and resistance gene profiles

All *V. paracholerae* genomes analysed lacked the major *V. cholerae* virulence determinants, CTX and TCP (Table S2), a feature also reported for another member of the Cholerae clade, *V. metoecus* [9]. However, segments of the Vibrio Pathogenicity Island-2 (VPI-2) containing the sialic acid metabolism cluster (*nan-nag*) were identified in 35 genomes, with the neuraminidase gene (*nan*H) detected in 32 of these. This pattern of modular fragmentation and retention of VPI-2 loci mirrors observations in *V. metoecus*, in which the *nan-nag* cluster was proposed to have originated through independent acquisition of VPI-2 islets [9]. The *tor* operon, involved in anaerobic respiration of trimethylamine N-oxide and known to enhance CTX production in *V. cholerae* [18], was also detected in a truncated form: five genomes contained *tor* genes, with two harbouring *tor*AC and three harbouring *tor*DR. These results indicate that *V. paracholerae* has undergone either partial acquisition or loss of virulence-associated genomic islands, suggesting divergence in ecological adaptation relative to *V. cholerae*.

Despite lacking the major cholera virulence factors, *V. paracholerae* harbours a broad repertoire of virulence-associated genes. The haemolysin (*hly*A) and thermolabile haemolysin (*tlh*) were nearly ubiquitous (220 out of 222 genomes), as were RTX toxin components (*rtx*B, *rtx*C and *rtx*D, present in 216, 217 and 218 genomes, respectively) (Table S2). The cholix toxin (*chx*A) was identified in 91 out of 222 genomes, consistent with previous observations [4]. The type VI secretion system, implicated in interbacterial competition and environmental fitness [19], was present in all genomes, whereas the type III secretion system (T3SS) was restricted to only two genomes (Table S2). The T3SS regions in these two genomes displayed ~97% coverage and identity to *V. cholerae* counterparts, suggesting recent horizontal acquisition likely facilitated by niche overlap between the two species. However, unlike T3SS islands in *V. mimicus* and *V. parahaemolyticus*, these loci lacked the *tdh* and *trh* toxins commonly associated with virulence [20].

Resistome analysis revealed between 4 and 17 ARGs per genome, with a median of five (Table S3). The most prevalent ARGs were *bla*CARB-9/9-like (*n*=113), *qnr*VC4/4-like (*n*=24), *qnr*VC5/5-like (*n*=13) and *bla*CARB-7/7-like (*n*=15), along with sporadic genes such as *qnr*VC7/7-like, *cat*B9-like, *aph*, *flo*R, *dfr*A6-like, *sul*, *qac* and *tet*(A/C) (Table S3). Given that *V. cholerae* carries non-mobilizing chromosomal platforms known as SIs, which are hypothesized as reservoirs of ARGs [21], we explored this aspect for *V. paracholerae*. By analysing the genomic context of each ARG, we identified flanking VCRs and/or class 4 integrase (*int*I4) signatures in 101 out of 222 genomes. ARGs confirmed within SIs included *bla*CARB-9/9-like (*n*=86), *bla*CARB-7/7-like (*n*=9), *qnr*VC4 (*n*=17), *qnr*VC4-like (*n*=4), *qnr*VC5 (*n*=2), *qnr*VC7-like (*n*=3), *cat*B9-like (*n*=4) and *dfr*A6-like (*n*=1) (Fig. 1). Additionally, some genomes contained multiple ARGs within their SI, with combinations such as *bla*CARB plus *qnr*VC or *cat*B9, and in some instances, duplicate copies of the same ARG allele (*bla*CARB or *qnr*VC). Indeed, several *bla*CARB alleles have recently been associated with VCRs in *V. cholerae*, suggesting their linkage to the *V. cholerae* SI [22], a pattern that is also supported by our findings for *V. paracholerae*. Additionally, the *qnr*VC4 and *qnr*VC5 alleles have previously been reported in association with VCRs [23], supporting our findings for *V. paracholerae*.

When compared with all known *bla*CARB alleles, the *bla*CARB variants identified in this study in SIs exhibited 98.96–99.89% nucleotide identity to *bla*CARB-7 or *bla*CARB-9, with 35 *bla*CARB-9-like sequences assignable to *bla*CARB-61. In fact, *bla*CARB-61 was also recently reported in an environmental *V. paracholerae* previously misidentified as *V. cholerae* [22]. A few amino acid substitutions were observed in specific genomes, including K201N in GCA_017169525, D58E in GCA_045017515 and GCA_028490905 and I32M in GCA_900538065 and GCA_003312095. The GCA_045016285 genome displayed a deletion affecting residue E80 in addition to the substitution K94Q, whereas GCA_008083255 contained a deletion of nucleotide A743, resulting in a frameshift that altered the downstream amino acid sequence from positions 250 to 288. Despite these changes, all *bla*CARB amino acid sequences retained the conserved motifs characteristic of penicillin-recognizing enzymes [22], indicating that these variants are likely functional.

The *qnr*VC variant alleles presented 98.33–98.94% identity to *qnr*VC4 (GCA_033803245.2, GCA_017169515.1, GCA_043002275.1 and GCA_045016285.1) and 99.85% identity to *qnr*VC7 (GCA_028490905.1). In addition, two *qnr*VC7-like sequences (GCA_048551175.1 and GCA_048551205.1) could be determined as *qnr*VC9. These nucleotide changes resulted in a few amino acid substitutions: GCA_033803245.2 presented H21 (Q21 in QnrVC4/5/7), while GCA_017169515.1, GCA_043002275.1 and GCA_045016285.1 presented D183 (E183 in QnrVC4/5/7). These substitution sites have not been described as essential for the enzymatic function of Qnr; therefore, only *in vitro* analyses could reveal any functional impact [24].

Regarding the *cat*B9 variants, four genomes (GCA_008083255.1, GCA_030710345.1, GCA_030718785.1 and GCA_049391525.1) harboured identical amino acid substitutions (V43A and N202Y), despite being isolated from different locations and time points (Table S1). Only one genome, GCA_027921665.1, carried a *dfr*A-like gene, which showed the highest similarity to *dfr*A46 but contained an H89N substitution.

As previously demonstrated, ARGs embedded in SIs of *V. paracholerae* can be transcriptionally active and functional [10]. Therefore, these findings support the role of *V. paracholerae* as an ancient and persistent reservoir of ARGs, paralleling the evolutionary dynamics observed in *V. cholerae*.

## Conclusion

An unsuspected set of genomes previously classified as *V. cholerae* (~1.8 %) was reclassified as *V. paracholerae*, underscoring the misidentification of this sister species in both clinical and environmental surveillance that can eventually impact the epidemiological analyses of cholera disease. *V. paracholerae* lineages have demonstrated long-term spatiotemporal persistence across diverse ecological niches, including human clinical cases, animals and natural environments, reflecting their broad adaptability. Moreover, several of these *V. paracholerae* genomes were linked to human infections, reinforcing their clinical and epidemiological relevance. Importantly, this study identified genetic markers that can be applied to distinguish *V. paracholerae* from *V. cholerae*, providing a practical tool for species identification and epidemiological surveillance.

## Supplementary material

10.1099/mgen.0.001605 Uncited Supplementary Material 1.

## Abbreviations

AF: alignment fraction
ARGs: antibiotic resistance genes
CTX: cholera toxin
dDDH: digital DNA–DNA hybridization
gANI: whole-genome Average Nucleotide Identity
SI: superintegron
TCP: toxin-coregulated pilus
T3SS: type III secretion system
VPI-2: Vibrio Pathogenicity Island-2
